# Socio-Economic and Environmental Factors Related to Spatial Differences in Human Non-Tuberculous Mycobacterial Diseases in the Czech Republic

**DOI:** 10.3390/ijerph16203969

**Published:** 2019-10-17

**Authors:** Helena Modrá, Vít Ulmann, Jan Caha, Dana Hübelová, Ondřej Konečný, Jana Svobodová, Ross Tim Weston, Ivo Pavlík

**Affiliations:** 1 Faculty of Regional Development and International Studies, Mendel University in Brno, Tr. Generala Piky 7, 613 00 Brno, Czech Republic; jan.caha@mendelu.cz (J.C.); dana.hubelova@mendelu.cz (D.H.); ondrej.konecny@mendelu.cz (O.K.); ivo.pavlik@mendelu.cz (I.P.); 2 Public Health Institute Ostrava, Partyzanske Nam. 7, 702 00 Ostrava, Czech Republic; vit.ulmann@zuova.cz; 3 IFCOR Klinicke Laboratore Ltd., Vinicni 235, 615 00 Brno, Czech Republic; j.svobodovab@gmail.com; 4 Department of Biochemistry and Genetics, La Trobe Institute for Molecular Science, La Trobe University, Science Dr, Bundoora, Melbourne VIC 3086, Australia; R.Weston@latrobe.edu.au

**Keywords:** mining, occupational exposure, *Mycobacterium chelonae*, heavy industry, mycobacteriosis, social deprivation

## Abstract

Non-tuberculous mycobacteria (NTM) are ubiquitous environmental bacteria that can induce pulmonary and non-pulmonary diseases in susceptible persons. It is reported that the prevalence of NTM diseases is increasing in developed countries, but this differs by regions and countries. NTM species distribution and the rate of diseases caused by NTM vary widely in the historical territories of Moravia and Silesia (Czech Republic). This epidemiologic study of NTM diseases covers the period 2012–2018, reviews isolates obtained from patients with clinical disease and investigates correlations with related socio-economic and environmental factors. Individual NTM patients were included only once during the studied period and results were presented as incidence rate per year. The most frequently isolated NTM meeting the microbiological and clinical criteria in the study were the *Mycobacterium avium-intracellulare* complex, followed by *Mycobacterium kansasii* and *Mycobacterium* *xenopi*. A previously described endemic incidence of *M.* *kansasii* in the Karviná district and *M.* *xenopi* in the Ostrava district was also observed in this study. The incidence of NTM patients in the whole studied territory was 1.10/100,000 inhabitants (1.33/100,000 in men and 0.88/100,000 in women). The annual incidence of lymphadenitis in children (≤5 years of age) was 2.35/100,000 of the population of children during the 7 year period but increased in the year 2018 to 5.95/100,000. The rate of human tuberculosis in the studied area was 1.97/100,000 inhabitants. The incidence of NTM pulmonary diseases correlated with a lower socio-economic status (*r* = 0.63) and a higher concentration of benzo[a]pyrene pollution in the air (*r* = 0.64).

## 1. Introduction

Non-tuberculous mycobacteria (NTM) are ubiquitous environmental bacteria commonly found in water biofilms, soils, dust, and sediments [1,2,3,4]. Some NTM species are opportunistic pathogens that are known to cause lung diseases and non-pulmonary infections in susceptible individuals [5]. An increase in the prevalence of NTM pulmonary diseases has been recorded in developed countries [6,7,8,9]. This increase has caused a substantial financial burden due to patient treatment and hospitalization [10]. Probable explanations for the increase in the frequency of clinically relevant isolates of NTM are longer life expectancy and the improvement in mycobacterial DNA isolation techniques and other improvements in laboratory diagnostics [2]. Significant geographic differences in the distribution of different NTM species in human clinical isolates were recently described worldwide [11,12,13]. The factors associated with this phenomenon are climate differences, population density, host-related factors (genetic background and occupational exposure) and the presence of a specific environmental niches [11,14,15,16].

Important risk factors of NTM pulmonary diseases are immunodeficiency and various structural lung diseases, such as chronic obstructive pulmonary disease (COPD), bronchiectasis, and cystic fibrosis [16,17]. The development of COPD and/or similar diseases is manifested by diminishing bronchial epithelium integrity. It can be connected to smoking and airborne pollution comprising particulate matter (PM) [18,19]. PM of varying size, origin and chemistry can absorb harmful pollutants such as polycyclic aromatic hydrocarbons (PAHs) that induce the production of reactive oxygen species (ROS) and inflammatory responses through the interaction with the aryl hydrocarbon receptor and the subsequent activation of cytochrome P450. These mechanisms make airborne pollution a potential risk factor for pulmonary tissue impairment and consequently could increase the risk of NTM pulmonary diseases in polluted urban and industrial areas [20,21]. Exposure to aerosol contaminated by NTM is another possible factor, causing NTM lung diseases in miners and metalworkers who may already have impaired lung alveoli as a consequence of a polluted occupational environment [15,22,23].

The historical territory of Moravia and Silesia in the Eastern part of the Czech Republic includes the Ostrava and Karviná districts—the most polluted areas in Central Europe from airborne dust with high concentrations of dust particles (PM_2.5_ and PM_10_) and benzo[a]pyrene, which is one of the most harmful PAHs [24,25]. Historically, the environment of these districts has been polluted as a result of mining activities and heavy industry, and although presently, heavy industry and black coal mining are in decline, air pollution in this area still continues [26]. Currently, stationary combustion sources are the major emitters of pollutants with geographical and weather conditions also playing a significant role in maintaining the high level of air pollution in this area. A high endemic incidence of infections caused by *Mycobacterium kansasii* has been reported in the Karviná district since 1968. A correlation between a higher incidence of *M. kansasii* diseases and employment in the mining industry in this district was described during the period 1968–1995 [27]. During the last two decades, the mining industry has been in decline and, consequently, the number of people employed in the mining industry has significantly reduced from 32,000 in 1995 to 12,500 in 2015 [28]; however, there continues to be an enhanced NTM incidence in this area (unpublished data from the Public Health Institute Ostrava, Czech Republic, 2019).

We hypothesize that air pollution and the worsening socio-economic situation of the resident population are co-contributing factors, resulting in a higher NTM incidence in some parts of the Czech Republic. To verify this hypothesis, three research aims were assessed: (1) to find differences in the geographic distribution of the total number of NTM disease incidence and individual NTM species in the studied area; (2) to analyze the relationship of selected demographic and socio-economic factors to the incidence of NTM-infected patients; and (3) to investigate a connection between air pollution indicators and NTM lung disease incidence. We also compared the age and sex of patients with human tuberculosis caused by *Mycobacterium tuberculosis* (MTB) and NTM diseases in order to find differences in these demographic parameters. We included data from a 7 year period (2012–2018) in our study.

## 2. Materials and Methods

### 2.1. Area Description and Study Design

**Figure 1:**
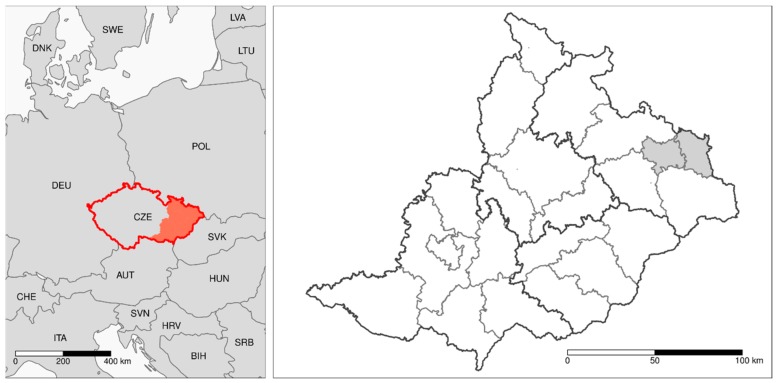
Left: location of the Czech Republic (CZE) in Central Europe and the studied area is colored in red. Right: individual districts (n = 22) within the studied area. The Karviná and Ostrava districts are shaded grey.

The Moravian and Silesian Regions (area 21,849 km^2^) of the Czech Republic (total area 78,867 km^2^) consist of 22 districts classified at the Local Administrative Unit (LAU) 1 level according to the European Union (Figure 1).

**Figure 2:**
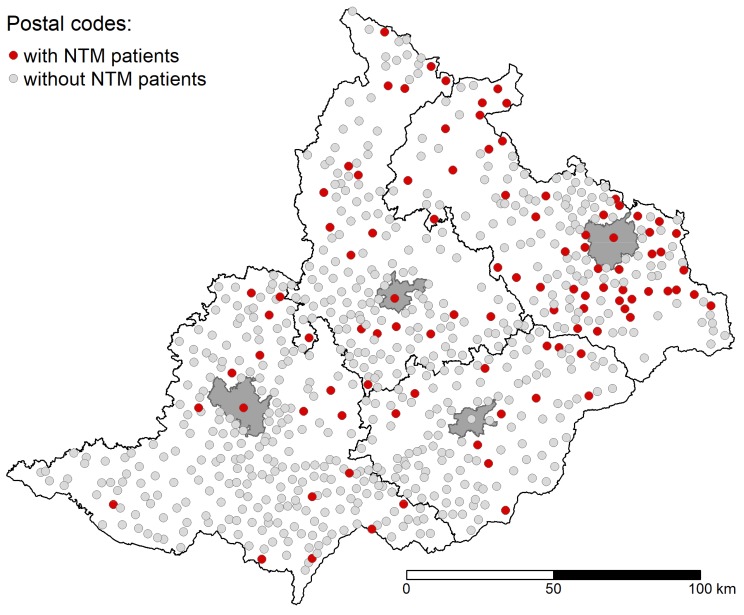
Postal codes within the studied area. Postal codes, from where non-tuberculous mycobacteria (NTM) patients originated are colored red.

The total population in the studied area varied from 3.620 mil in 2012 to 3.604 mil in 2018. Within the studied area, territorial units (n = 710) were defined according to the postal codes of the home address of the patient from which the NTM was isolated and this was used to construct a study area map (Figure 2). The size of the population per territorial units ranged from 151 to 377,973 persons (median 1971) from 2012 to 2018.

To assess the relationship between NTM incidence and other factors (socio-economic indicators, benzo[a]pyrene, PM_2.5_ and PM_10_ air pollution), the NTM incidence data at the postal code level were integrated with data on other factors which were collected at the district level (LAU 1). The number of patients was adjusted (normalized) to the population of the given municipality using data provided by the Czech Statistical Office in the given year [29].

### 2.2. Data Source

Recorded identification of NTM human isolates was obtained from the Public Health Institute Ostrava (Czech Republic). Mycobacterial isolates were classified as the causative agent of disease according to the American Thoracic Society (ATS)/the Infectious Diseases Society of America (IDSA) microbiologic criteria as well as stricter microbiological criteria (multiple isolation of the same NTM species from respiratory samples, acid-fast stain-positive results, sole NTM isolation from bioptic tissues or sterile body fluids); and clinical criteria (significant progressive changes on a chest skiagram and/or HRCT; high-resolution computed tomography), nodular-bronchiectatic or cavernous lesions in association with a chronically worsened overall condition, and systemic or skin manifestation. The types of NTM diseases were divided into two groups: NTM pulmonary and NTM non-pulmonary diseases.

Individual NTM patients were included only once during the studied period and results were presented as an average incidence (new cases per population) per year.

The selected demographic indicators (sex and age) and socio-economic factors for every year (2012–2018) were obtained from the database of the Czech Statistical Office [29]. Four socio-economic indicators were selected: the crude divorce rate, the net migration rate (the difference between the number of people coming into an area and the number of people leaving an area throughout the year), the share of the paid housing allowance, and the percentage of unemployment. Based on these indicators an index of social deprivation was generated per region (LAU1).

The average concentrations of PM_2.5_, PM_10_ and benzo[a]pyrene in the air during the period 2012–2016 were obtained from the Czech Hydrometeorological Institute [30].

### 2.3. NTM Identification

All potential NTM isolates were first identified by macroscopic and Ziehl-Neelsen microscopic examinations. Mycobacterial isolate identification was carried out by AccuProbe Test (Hologic, San Diego, USA), by PCR with reverse hybridization on cellulose strips, GenoType Mycobacterium CM/AS assays (Hain Lifescience, Nehren, Germany) and/or by sequencing genes encoding the 16S ribosomal RNA subunit (Applied Biosystems Genetic Analyzer ABI3130 series, Thermo Fisher Scientific, Waltham, MA, USA).

### 2.4. Data Analysis and Statistical Methods

The incidence of each NTM species in patients every year was calculated and then presented as average annual incidence in the studied period 2012–2018. Demographic and socio-economic data for the year 2018 were not assessed due to the unavailability of this information on this date. Data on the four selected socio-economic indicators averaged over 6 years period (2012–2017) were converted to relative values, normalized with the Min–Max method and aggregated into an index of social deprivation [31].

The correlated data were assessed using the Pearson’s correlation coefficient. The comparisons of incidence distributions through the years were performed using the Kruskal–Wallis Rank Sum Test. All the data processing and statistical testing were performed in R [32], with the use of the packages *dplyr* [33] and *ggplot2* [34] for data manipulation and visualization, and the packages *sf* [35] and *tmap* [36] for spatial data manipulation and visualization in the form of maps.

## 3. Results

In total, 2176 mycobacterial isolates belonging to 32 NTM species were recorded during the 7 year period. The identification of 66 (3.03%) clinical isolates was unsuccessful (Table 1). A total of 303 (13.92%) NTM isolates (one per patient) were identified as causative agents of disease according to ATS/IDSA microbiological and clinical criteria and consequently included in this study. None of the NTM or MTB patients who underwent human immunodeficiency virus (HIV) testing were positive.

*M. avium-intracellulare* complex (*MAI*C) members and ten NTM species meeting the microbiological and clinical criteria were encountered in this epidemiological study. Two NTM isolates from patients could not be identified. The most frequently isolated NTM were the *MAI*C members, followed by *M. kansasii*, and *Mycobacterium xenopi.* These NTM species accounted for almost 90% of all isolates from patients. Rapidly growing NTM (*Mycobacterium chelonae*, *Mycobacterium fortuitum*, *Mycobacterium abscessus*, and *Mycobacterium mucogenicum*) caused 6.6% of NTM diseases overall (Table 1).

**Figure 3:**
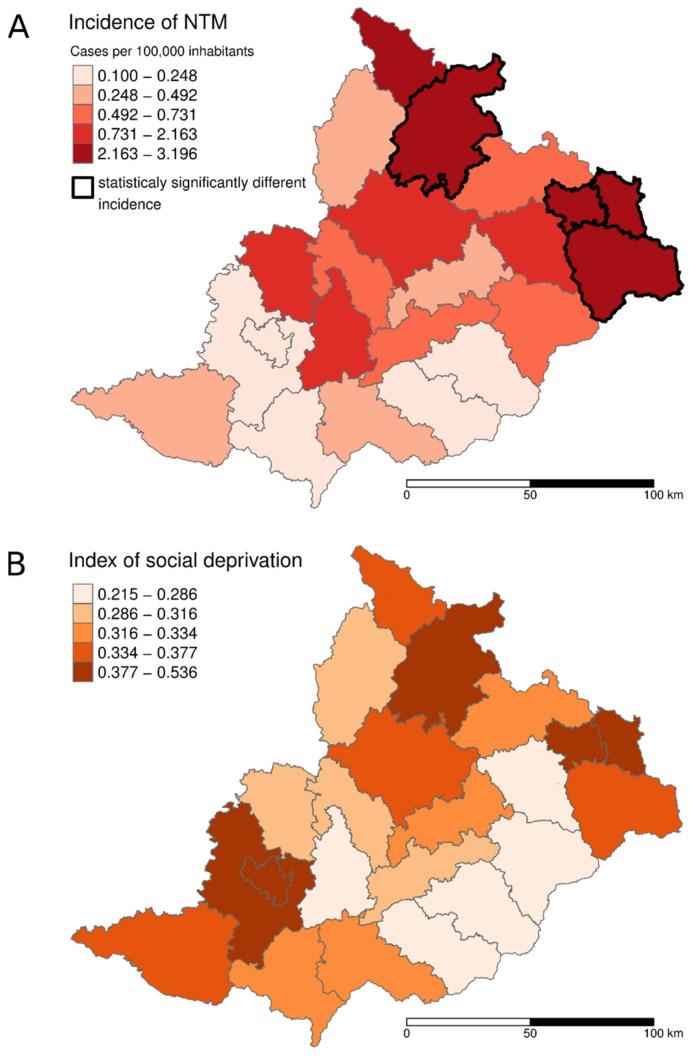
(**A**) Average incidence of NTM pulmonary diseases; (**B**) Index of social deprivation in the districts of the studied area during the period 2012–2018.

**Figure A1:**
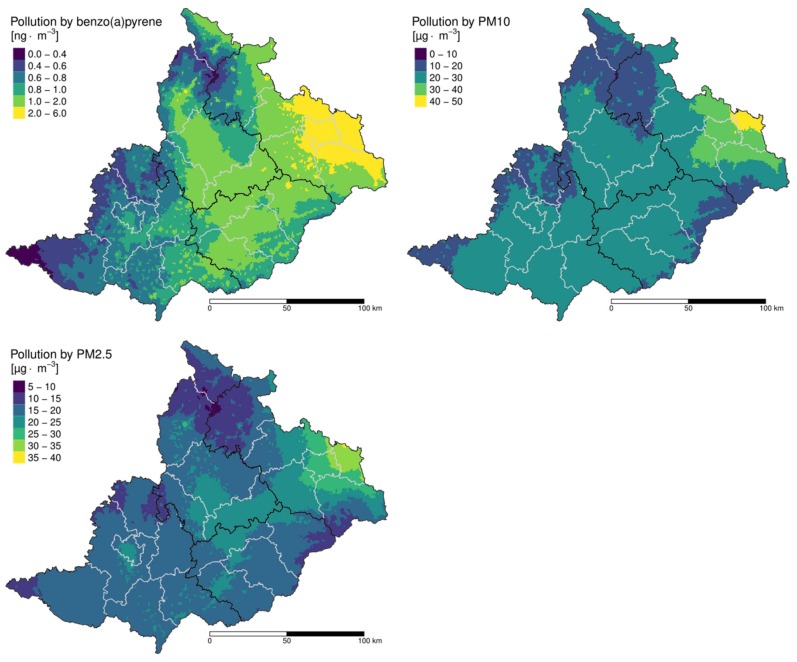
Five-year (2012–2016) average concentrations of benzo[a]pyrene, PM_10_ and PM_2.5_ in the air in the studied regions based on the data from the Czech Hydrometeorological Institute [30].

The average incidence of NTM patients in the studied area was 1.10/100,000 inhabitants (1.33/100,000 in men and 0.88/100,000 in women) during the 7 year period. *MAIC* members’ pulmonary diseases were more frequent in women than men. However, *M. kansasii* and *M. xenopi* were significantly more often diagnosed in men (*p* < 0.05). Compared to the average, a statistically higher incidence of NTM in adult patients was found in four districts (*p* = 0.0012) located in the north part of the studied area: Bruntál, Jeseník, Karviná, and Ostrava districts (Figure 3A; Table A1). The correlation between NTM pulmonary disease incidence and the index of social deprivation was 0.63. A visualization of the level of social deprivation is shown in Figure 3B. NTM pulmonary disease incidence correlated with a higher than average benzo[a]pyrene concentration in the air (*r* = 0.64). The correlations between particle pollutions PM_2.5_ and PM_10_ were 0.41 and 0.42, respectively. The maps of benzo[a]pyrene, PM_2.5_ and PM_10_ concentrations in the air are available in Figure A1.

**Figure 4:**
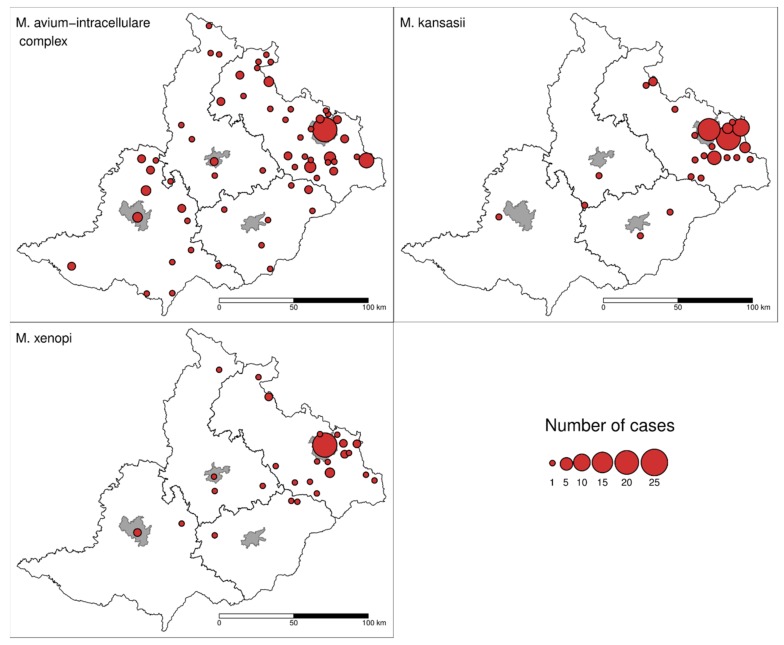
Location and number of patients with the three most frequent NTM species in the studied area during the period 2012–2018.

The occurrence of patients with *MAI*C members, *M. kansasii* and *M. xenopi* pulmonary diseases are shown in Figure 4. A significantly higher incidence of *M. kansasii* (*p* = 0.0006) was recorded in the Karviná district (1.90/100,000) compared to all other districts. A higher incidence was recorded in men (2.86/100,000) compared to women (0.99/100,000) in this district. A statistically higher incidence of disease caused by *M. xenopi* (*p* = 0.03) was detected in the Ostrava district (1.01/100,000; Table A1).

**Figure 5:**
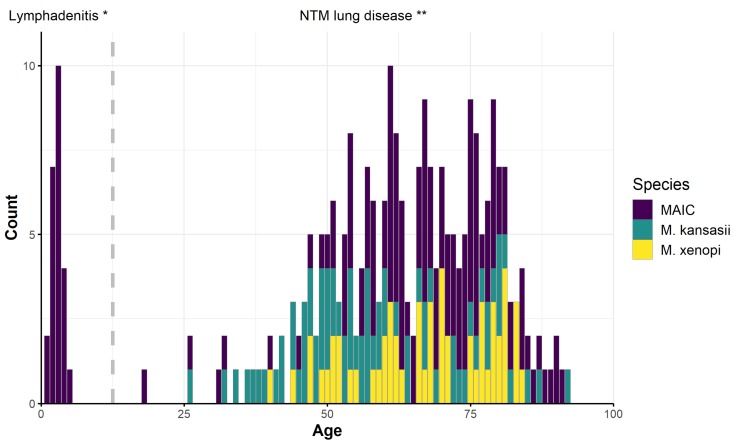
Type of the diseases, number of patients with the most frequent NTM complex and species, and the number of cases by age. * 100% of patients under 5 years of age suffered from cervical lymphadenitis; ** 98.02% of patients older than 18 years suffered from NTM lung diseases in the period 2012–2018. *MAI*C: *M. avium intracellulare* complex.

The distribution of the three most frequently recorded NTM causative agents (*MAI*C members, *M. kansasii*, and *M. xenopi*) in patients by age is shown in Figure 5. All 26 NTM patients under the age of 5 years had cervical lymphadenitis caused by *MAI*C members. In patients over 18 years of age, 98% of the cases of NTM disease were pulmonary infections. Non-pulmonary diseases were recorded in six adult patients: two cases were caused by *M. chelonae* and *M. marinum* and one case each by *M. chimaera* and *Mycobacterium vulneris*. *M. chelonae* was isolated from the eye of a patient who suffered from keratitis after surgery and the patient suffered from a periodontitis abscess. *M. marinum* and *M. vulneris* were isolated from cutaneous lesions of two patients, and *M. chimaera* was a causative agent of pleurisy after surgery in one patient.

The rate of lymphadenitis caused by *MAI*C members in children (<5 years) was 2.35/100,000 during the studied period; the rate was especially high in the final year of the study, at 5.92/100,000.

**Figure 6:**
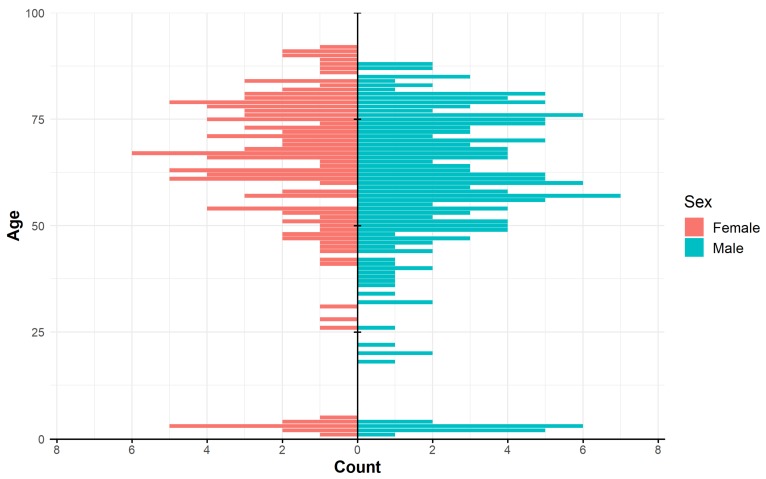
Histogram of NTM diseases by age and sex in the period 2012–2018.

**Figure 7:**
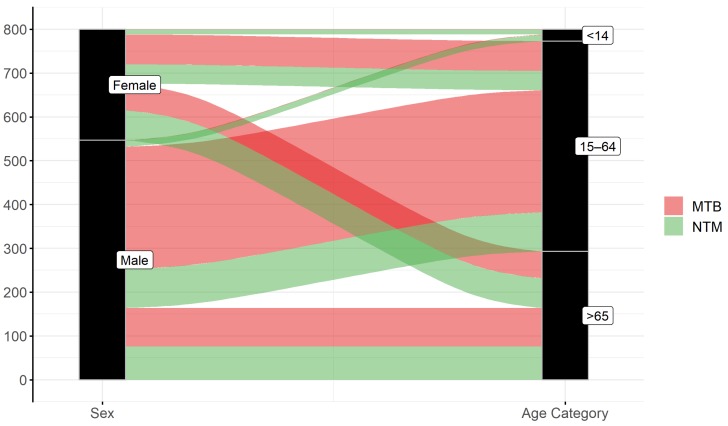
Sankey diagram: Comparison of the number of human tuberculosis (MTB, red color) and non-tuberculous mycobacteria (NTM, green color) patients with respect to gender (left) and age (right) in Moravian and Silesian territories of the Czech Republic (2012–2018). Males in the category 15–64 years suffered from MTB more often than NTM, but males 65 years and over had equal incidence of MTB and NTM. Total number of both MTB and NTM patients was lower in females in comparison to males.

The median age of all NTM patients was 66.0 years. The median age of patients with *MAI*C members and *M. xenopi* infections was 68.0. *M. kansasii* infections were more frequently diagnosed in middle age patients (median age 56.0 years). The median age of children with lymphadenitis was 3.0 years. The number of patients, as well as their age and sex are shown in Figure 6. The overall incidence of MTB pulmonary diseases was 1.97/100,000. A higher number of MTB compared to NTM patients was found in adult men (age 15–64 years). Roughly, an equal number of MTB and NTM diseases were recorded in men and women older than 65 years (Figure 7).

## 4. Discussion

The reported incidence of clinically relevant NTM can differ depending on the methodology used to count NTM isolates, causing a discrepancy between isolation rate and actual disease diagnosis—specifically, whether the particular methods take into account multiple NTM isolates per patient, latent infections and transient presence of the NTM without clinical relevance [16]. In European countries, the prevalence of NTM pulmonary diseases meeting ATS/IDSA and stricter microbiological criteria make up only 5.5%–26.0% of the total number of NTM clinical isolates [12,37,38,39,40,41,42]. These findings are in accordance with the results of our study, where we confirmed that less than 14% of total NTM isolates were responsible for clinical disease (Table 1).

Environmental NTM is well adapted to outdoor survival in extreme conditions and their abundance in environment is influenced by environmental and geochemical characteristics [43,44,45,46]. NTMs are opportunistic pathogens with the potential to colonize tissues of immunocompromised or “susceptible” persons, especially from sources with higher numbers of infective units or sources with greater frequencies of human exposure [46,47,48]. Defensive mechanisms of healthy humans are capable of eliminating primary infection even in immunocompetent individuals in most cases. Natural self-cleaning/healing lung functions (debacilization), effective expectoration and ciliary transport may be the most effective elements of host defense against environmental NTM. The primary infectious dose of most NTM infections in immunocompetent and physiologically healthy individuals may even be eliminated by naive alveolar macrophages. Signaling cytokine pathways and the activation of macrophages are initiated within hours after NTM infection in comparison with the long delay seen in MTB infections [49].

The obligate pathogenic *M. tuberculosis* complex members and *M. leprae* have developed mechanisms (effective blockage of phagosome fusion with lysosomes by sulpholipide-1, deregulation of cytokine signaling pathways, and dormancy) to evade the host immune system, which allow them not only to survive phagocytosis but also to activate intracellular proliferation [50]. Compared to MTB, NTMs are equipped with much less effective virulence factors (cell wall structure, etc.) and their survival in the host organism depends on the degree of adaptability of that particular NTM species or even isolate. Factors for overcoming the interaction with the immune system vary significantly. The most basal mechanisms are wide range temperature tolerance and ability to produce compact cell formations: cording and clustering, as seen in *M. kansasii* and *M. xenopi*, or biofilm formation as seen in *MAIC* members [51]. Enzymatic and structural adaptations of the cell wall of NTM species associated with animal diseases (i.e., fish tank granuloma caused by *M. marinum*) can confer an advantage in the colonization of damaged human skin [52]. Pathological lesions in the pulmonary structure based on an underlying disease can create niches that allow further growth of NTM. Bronchiectasis, bronchial obstruction (asthma, COPD), fibrous tissue (silicosis, coniosis, other granulomatosis, and prior MTB) as well as cystic fibrosis can result in permanent deposition of air pollution, persistent mucus, and may also alter the effectiveness of neutrophils [53,54,55]. Some NTM can cause latent infections in lung tissue, and during the latency period, NTM can be isolated from expectorated sputum even when there are no apparent clinical symptoms. Long-term latency or inactivity of NTM and the graduating colonization of affected lung tissue is to be assumed. The patients in this period produce NTM cells in the expectorated sputum, but without significant apparent symptoms. Sufficient moisture and a microaerophilic environment in impaired areas of lungs can enable mycobacteria to multiply, leading to immune system hyperactivity and the progression of symptomatic disease [16].

The species distribution of NTM patient isolates in European countries was described recently [11]. However, this study did not include results from the Czech Republic. In this study, there was a significant difference between the geographical distribution of NTM isolation frequency and NTM diseases. If isolates of *Mycobacterium gordonae* and *Mycobacterium fortuitum*, which have a low clinical relevance anyway, are omitted, the *MAI*C members predominate in the North and West European regions [11,39,56]. This is different to what is seen in Eastern European countries, where *M. kansasii* is the predominant NTM in Poland and Slovakia and *M. xenopi* is the most isolated species in Hungary and Croatia [11,12,57]. These results correlate with our observations, where the north part of the studied area (near the border with Poland) has a higher prevalence of *M. kansasii*, and in the remainder of the studied area, *MAIC* is more prevalent (Figure 4). Rapidly growing NTM, common in the UK and Greece [11], formed less than 7% of the total NTM patients in our study (Table 1). *Mycobacterium malmoense* occurs mostly in Northern Europe [11,58]. In this study, it was recorded in six patients living in the north part of studied area (data not shown).

The annual incidence of children infected with *MAI*C members in our study (2.35/100,000 children’s population) is similar to estimates reported in other European countries such as the Netherlands [59] and Germany [60]. *MAIC* complex members have been known to be causative agents of lymphadenitis in children since the 1970s [61]. An increase in NTM disease incidence was documented in France after the discontinuation of mandatory *Bacillus Calmette Guérin* (BCG) immunization in 2007 [62]. We did not observe an increasing incidence of NTM disease during the period 2012–2017, although a sharp increase (5.92/100,000 population) was recorded during the year 2018.

The mean age of adult NTM patients in our study was similar to other European countries. The median age of NTM adult patients in Central Greece (2004–2006), Croatia (2006–2013), and Denmark (2015) was 66.1, 67.0, and 68.2 years, respectively [38,39,63]. The age of adult patients with *M. kansasii* pulmonary diseases was lower than the age of patients with *M. xenopi* and *MAI*C pulmonary diseases in other similar studies [63,64,65] which is in accord with the findings of this study (Figure 5). Generally, adult NTM patients are more often male [42,63,64,66] with the exception of *MAI*C pulmonary diseases, which are often diagnosed in older women [65,67].

It is thought that elderly patients are exposed to NTM in indoor environments through contaminated tap water. On the one hand, a link between potable water NTM isolates and human diseases using PCR methods has been recorded in *MAIC* members and *M. chimaera* infections. On the other hand, very few strains of *M. kansasii* isolates from tap water matched disease isolates [68,69]. Shower usage by elderly individuals is less frequent, which can lead to long term water stagnation and may allow NTM colonization of household plumbing. This fact in combination with other comorbidities may be one of the reasons why NTMs are more frequently isolated in elderly patients than the general population [70,71,72]. Higher NTM prevalence may also be linked to water supply practices, specifically chlorination of old municipal water supplies, which can concentrate the NTM in biofilms [73]. The importance of this factor is supported by the finding of a higher abundance of NTM in showerhead biofilms in USA domestic water systems compared to Europe, where a lower chlorine concentration is used during water treatment [74].

Pulmonary disease caused by *M. kansasii* has been endemic in the Karviná district since the 1970s [75]. During the period 1984–1989, the rate of NTM incidence in this district fluctuated between 13.5 and 17.6/100,000 inhabitants, which was much higher than the national average [75]. Subsequently, in the 1990s, there was a decrease in the prevalence of NTM patients being recorded; this coincided with and is probably related to mining restrictions that came into practice after the “velvet revolution” in 1989 [27]. The majority of cases were described in men (81%) during the period 1968–1995 and a link with the mining industry has been established in 56.4% of patients. Currently, there is still a statistically significantly higher incidence of *M. kansasii* pulmonary diseases in men from the Karviná district, at 2.86/100,000 of the population compared to the average of the entire studied area (0.44/100,000 population; Table A1). We also observed a higher incidence of *M. kansasii* pulmonary diseases from the Karviná district (0.99/100,000) in women compared to the overall incidence (0.16/100,000). This suggests that employment in the mining industry (which predominantly employs males) is not the only risk factor for *M. kansasii* infection as described in previous studies [27,76]. A relatively high level of *M. kansasii* detection in tap and surface water in the Karviná district could represent a possible risk factor [27,77]. However, a link between potable water isolates of *M. kansasii* and clinical isolates has not been confirmed up to now and point source contamination may occur from an alternative environmental source [69]. A higher incidence of *M. kansasii* infections has also been recorded in other regions with heavy industry and mining [76]. The higher mean age (56.0) of men with *M. kansasii* infection in the period 2012–2018 described in our study compared to the average age of 50.1 years during the period 1984–1989 [75] is probably the consequence of the aging of former miners. The mining industry in the Karviná district employs significantly less people then previously but former mineworkers are still at risk of occupational pneumoconiosis (unpublished data).

NTM can survive and persist in household and industrial plumbing systems due to the ability of the bacteria to grow at a low nutrient concentration, associate in biofilms, and chlorine resistance [3,78]. The high correlation of *MAIC* detected in household water with high *MAIC* disease incidence may be a reflection of the large number of water-based studies that have been completed [68]. A possible exposure route is aerosol inhalation during personal hygiene and/or aspiration of NTM contaminated water [6,79]. Our study could not confirm this because environmental isolates were not analyzed and compared to clinical isolates. The practice of screening households of patients with NTM diseases to confirm the presence of an NTM contamination is not yet conducted in the Czech Republic. Establishing this practice, especially in the case of pediatric patients or patients with a long history of NTM infection would be desirable. The availability and effectiveness of methods to accurately type isolate strains are limited presently. The most commonly used methods are Restriction Fragment Length Polymorphism (RFLP) with Pulsed-Field Gel electrophoresis (PFGE), Mycobacterial Interspersed Repetitive Units – Variable Number of Tandem Repeats (MIRU-VNTR) and Whole Genome Sequencing (WGS). Linking strains isolated from a patient’s clinical material with those isolated from environmental sources is difficult using these methods. The possibility of the restructuring of the genome of NTM species as a reaction to stress and adaptations associated with colonization of a new patient’s tissue environment makes this even more complicated [80,81,82,83]. A long period from infection and colonization to disease development may also affect differences in the genetic profile of the NTM isolates from patients compared to those from the environment [81]. Despite these difficulties, a study by Kaustova et al. (1993) showed a connection between *M. xenopi* human isolates and contaminated household water in the Ostrava district in a study covering the period 1990–1991 [84].

A lower income and socio-economic status are connected with higher morbidity, disability, and mortality [85,86]. In addition, some known risk factors for NTM pulmonary diseases (heavy smoking and alcohol misuse) are connected to worsened socio-economic status. Some of the northern parts of the studied area, which includes the Ostrava and Karviná districts, are classed as having lower socio-economic status [87]. However, data about smoking and alcoholism were only available at region level (NUTS 3, *Nomenclature des Unites Territoriales Statistiques*) and consequently a direct comparison of these factors with our results was not possible. For this reason, we used an indirect indicator (index of social deprivation) of socio-economic status. A deeper social deprivation in the north part of the studied area including the Ostrava and Karviná districts as well as a statistically higher incidence of NTM pulmonary diseases was evident in our study (Figure 3A,B). Our findings correspond to the results of other studies conducted in developed countries. A spatial epidemiological analysis of NTM infections in connection with socio-economic attributes was carried out by Chou, et al. [88] in Queensland, Australia. The risk of *M. kansasii* diseases was estimated to decrease with an increase in the earning level of a population. Similarly, a higher incidence of *M. kansasii* pulmonary diseases in patients with a lower median income was described in Northern California, USA [89]. It is essential in this context to state, that all types of factors—host susceptibility, environmental and socio-economic factors (median income level)—must be considered during the evaluation of NTM disease risk [44].

Although the socio-economic situation and environmental pollution levels are important predisposition factors of MTB prevalence [90,91,92], the influence of air pollution on NTM pulmonary infections has not been studied yet. Air pollution consists of various gases and particles. Air particles smaller than 10 µm in diameter deposited in the respiratory tract can cause serious adverse effects on health due to the potential of being biologically active [93]. The coarse fraction of air pollution (PM_2.5–10_) can penetrate into the upper airways, while the fine fraction (PM < 2.5) can be deposited in the lung alveoli—both of which lead to several pulmonary pathological processes and an increased risk of respiratory infections [20,94]. The level of adverse health effects of PM is also influenced by particle-associated contaminants, such as heavy metals, polychlorinated dibenzo-p-dioxins, and PAHs [95,96]. Most particulate phase PAHs are adsorbed onto PM_2.5_. Benzo[a]pyrene is a highly carcinogenic agent used worldwide as a criterion for air pollution measurement [97,98,99]. Both PM_2.5_ and benzo[a]pyrene induce markers of oxidative stress through the aryl hydrocarbon receptor (AhR) pathway and consequently increase the risk of COPD [18]. Thus, air pollution through pulmonary impairment and lung disease increases the risk of NTM pulmonary infection caused by *M. kansasii,* and this is evident in our study (Figure 4, Table A1).

The Czech Republic has a low MTB incidence (4.9/100,000 population) according to the ECDC European Centre for Disease Prevention and Control (ECDC)/WHO report from 2016 [100]. The higher rate of MTB documented in the whole territory of the Czech Republic compared to the studied area (1.97/100.000) was due to the high MTB rate in the population of Prague—the capital city of the Czech Republic [101]. The rate of MTB was higher than NTM in the age group 15–64 in both men and women, and a similar incidence of MTB and NTM was recorded in the age group >65 (Figure 7).

The interpretation of results in our study is limited by not knowing each patient´s predisposing conditions and the analysis of water samples from plumbing systems suspected of NTM contamination. Furthermore, some clinical isolates (estimated to be less than 5 per year) sampled in the studied area were examined in alternative laboratories and could not be included in our results. However, this is estimated to be less than 2% of the isolates per year.

## 5. Conclusions

Our study complements the results of other European studies researching the distribution of different NTM species in humans and the incidence of NTM diseases. Our results show the essential differences between NTM human isolates and NTM diseases in both, the total number of cases and the NTM species spectrum. The incidence of NTM pulmonary diseases caused by different NTM species significantly differed even in the small geographic units (districts) defined in our study. In terms of NTM ecology, it appears that *M. xenopi* and *M. kansasii* diseases are endemic in the northern parts of the studied area. Our study indicates that lung diseases connected to air pollution, occupational exposure and a worsened socio-economic situation could lead to an increase in the risk of NTM pulmonary diseases. Future research focused on the examination of NTM isolates from the environment in the studied area would need to match clinical and environmental isolates and determine more specific environmental sources of NTM.

## Figures and Tables

**Table 1:** Species distribution in human isolates of non-tuberculous mycobacteria (NTM) in Moravian and Silesian territories (Czech Republic) during the years 2012–2018. NTM species in the column are ordered by the highest number of causative agents of the diseases. (Numbered NTM species were clinically important.)

No.	NTM Species	Total Individual Patient Isolates n (%)	Number of Isolates from Patients that Met ATS Criteria ^†^ n (%)
1.	*MAI*C *	386 (17.74)	143 (47.19)
2.	*Mycobacterium kansasii*	134 (6.16)	72 (23.76)
3.	*M. xenopi*	792 (36.40)	55 (18.15)
4.	M. chelonae **	41 (1.88)	7 (2.31)
5.	*M. malmoense*	10 (0.46)	6 (1.98)
6.	M. fortuitum **	210 (9.65)	6 (1.98)
7.	M. abscessus **	12 (0.55)	4 (1.32)
8.	M. mucogenicum **	45 (2.07)	3 (0.99)
9.	*M. marinum*	2 (0.09)	2 (0.66)
10.	*M. chimaera*	3 (0.14)	2 (0.66)
11.	*M. vulneris*	1 (0.05)	1 (0.33)
	*M.* sp.	66 (3.03)	2 (0.66)
	Subtotal	1702 (78.22)	303 (100)
	*M. gordonae*	341 (15.67)	0
	M. smegmatis **	55 (2.53)	0
	*M. celatum*	22 (1.01)	0
	M. goodie **	12 (0.55)	0
	*M. lentiflavum*	10 (0.46)	0
	M. peregrinum **	8 (0.37)	0
	M. neoaurum **	5 (0.23)	0
	*M. arupense*	3 (0.14)	0
	*M. scrofulaceum*	3 (0.14)	0
	Others ^#^	15 (0.69)	0
	Subtotal	474 (21.78)	0 (0)
	Total number	2176 (100)	303 (100)

* *Mycobacterium avium-intracellulare* complex, ** Rapidly growing NTM, and ^#^ NTM species in number ≤2. ^†^ each patient was included only once during the studied period.

## Appendix A

**Table A1:** The incidence of NTM diseases per districts in the studied territory during the years 2012–2018.

District	NTM			*MAIC*			*M. kansasii*			*M. xenopi*		
	Total	Female	Male	Total	Female	Male	Total	Female	Male	Total	Female	Male
Blansko	1.058	0.783	1.341	1.058	0.783	1.341	0	0	0	0	0	0
Brno-city	0.227	0.219	0.234	0.113	0.073	0.157	0	0	0	0.075	0.146	0
Brno	0.131	0.130	0.131	0	0	0	0.065	0	0.131	0	0	0
Bruntál	3.196	3.000	3.399	1.828	1.799	1.857	0.455	0	0.920	0.459	0.294	0.622
Břeclav	0.247	0.486	0	0.247	0.486	0	0	0	0	0	0	0
Frýdek-Mistek	2.479	2.507	2.451	1.272	1.847	0.680	0.671	0.264	1.091	0.402	0.265	0.544
Hodonín	0.369	0.183	0.561	0.369	0.183	0.561	0	0	0	0	0	0
Jeseník	2.186	1.445	2.937	1.091	1.445	0.733	0	0	0	0.367	0	0.739
Karviná	2.798	1.427	4.219	0.276	0.218	0.337	1.904	0.987	2.856	0.450	0.112	0.800
Kroměříž	0.539	0	1.103	0.134	0	0.274	0	0	0	0.135	0	0.276
Nový Jičín	2.072	2.043	2.102	1.036	1.115	0.956	0.282	0.186	0.382	0.376	0.370	0.382
Olomouc	0.733	0.475	1.005	0.244	0.357	0.125	0.062	0	0.126	0.123	0	0.253
Opava	0.728	1.111	0.330	0.567	0.794	0.330	0.081	0.158	0	0.081	0.158	0
Ostrava	2.770	1.877	3.719	1.012	1.194	0.818	0.704	0.341	1.088	1.011	0.339	1.722
Prostějov	0.526	0	1.078	0.263	0	0.539	0.132	0	0.270	0	0	0
Přerov	0.434	0.212	0.664	0.110	0	0.223	0	0	0	0.109	0	0.222
Šumperk	0.470	0.231	0.718	0.117	0.231	0	0	0	0	0	0	0
Uh. Hradiště	0.100	0	0.204	0.100	0	0.204	0	0	0	0	0	0
Vsetín	0.695	0.978	0.403	0.398	0.783	0	0.099	0	0.202	0.198	0.194	0.201
Vyškov	0.945	1.249	0.633	0.473	0.936	0	0	0	0	0.156	0	0.316
Zlín	0.223	0.146	0.304	0.149	0	0.304	0.075	0.146	0	0	0	0
Znojmo	0.252	0.497	0	0.252	0.497	0	0	0	0	0	0	0
Studied area overall	1.096	0.869	1.333	0.467	0.535	0.396	0.285	0.163	0.436	0.218	0.152	0.331

NTM = non-tuberculous mycobacteria; *MAIC* = *Mycobacterium avium-intracellulare* complex.
